# The Properties of Binding Sites of miR-619-5p, miR-5095, miR-5096, and miR-5585-3p in the mRNAs of Human Genes

**DOI:** 10.1155/2014/720715

**Published:** 2014-08-04

**Authors:** Anatoly Ivashchenko, Olga Berillo, Anna Pyrkova, Raigul Niyazova, Shara Atambayeva

**Affiliations:** National Nanotechnology Laboratory, Al-Farabi Kazakh National University, Almaty 050038, Kazakhstan

## Abstract

The binding of 2,578 human miRNAs with the mRNAs of 12,175 human genes was studied. It was established that miR-619-5p, miR-5095, miR-5096, and miR-5585-3p bind with high affinity to the mRNAs of the 1215, 832, 725, and 655 genes, respectively. These unique miRNAs have binding sites in the coding sequences and untranslated regions of mRNAs. The mRNAs of many genes have multiple miR-619-5p, miR-5095, miR-5096, and miR-5585-3p binding sites. Groups of mRNAs in which the ordering of the miR-619-5p, miR-5095, miR-5096, and miR-5585-3p binding sites differ were established. The possible functional and evolutional properties of unique miRNAs are discussed.

## 1. Introduction

MicroRNAs (miRNAs) participate in the regulation of the expression of protein-coding genes at the posttranscriptional stage [1]. miRNAs, as a part of the RNA-induced silencing complex, bind to mRNAs and interfere with translation or promote mRNA destruction [2]. The study of the properties of miRNAs and their influences on the expression of the genes that participate in all key cellular processes of cells was established in the last 20 years. The actions of miRNAs on the cell cycle [3], apoptosis [4], differentiation [5], and growth and development in plants [6] and animals [7] have been shown. Connections between miRNA expression and the development of various diseases have been established. miRNA concentrations change in cancer [8] and cardiovascular diseases [9]. Metabolic disturbances necessarily change miRNA concentrations in cells [10]. It is possible to normalize some processes using miRNAs [11]. The aforementioned roles do not encompass the full list of the biological processes in which miRNAs participate, which proves the importance of their biological functions.

Despite the appreciable successes in the study of miRNA properties, there are obstacles to establishing the target genes of miRNAs. Normally, one miRNA interacts with the mRNA of one gene. However, there are miRNAs that bind to many mRNAs, and one mRNA can be the target of many miRNAs. These circumstances significantly complicate the study of the properties of miRNAs and their diagnostic and medical applications. There are more than 2,500 miRNAs in the human genome, and they are thought to act on 50% or more of genes. It will be difficult to draw unique conclusions about the participation of miRNAs in specific biological processes, and until those conclusions can be drawn, the connections between the majority of miRNAs and their target genes will remain unknown.

Recently, we found a set of unique miRNAs that have hundreds of target genes and bind to mRNAs with high affinity. The binding sites unique to miRNAs are located in the 5′-untranslated regions (5′UTRs), the coding domain sequences (CDSs) and the 3′-untranslated regions (3′UTRs) of mRNAs. In present work, we studied some unique miRNAs that bind to the mRNAs of several hundred human genes.

## 2. Materials and Methods

The human gene mRNAs were taken from GenBank (http://www.ncbi.nlm.nih.gov/) using Lextractor002 script (http://sites.google.com/site/malaheenee/software). The nucleotide sequences of human miR-619-5p, miR-5095, miR-5096, and miR-5585-3p were taken from the miRBase site (http://mirbase.org/).

The target genes for the tested miRNAs were revealed using the MirTarget program, which was developed in our laboratory. This program defines the following features of binding: (a) the origin of the initiation of miRNA binding to mRNAs; (b) the localization of miRNA binding sites in the 5′-untranslated regions (5′UTRs), the coding domain sequences (CDSs), and the 3′-untranslated regions (3′UTRs) of the mRNAs; (c) the free energy of hybridization (Δ*G*, kJ/mole); and (d) the schemes of nucleotide interactions between the miRNAs and the mRNAs. The ratio Δ*G*/Δ*G*_*m*_ (%) was determined for each site (Δ*G*_*m*_ equals the free energy of an miRNA binding with its perfect complementary nucleotide sequence). The miRNA binding sites located on the mRNAs had Δ*G*/Δ*G*_*m*_ ratios of 90% or more. We also noted the positions of the binding sites on the mRNA, beginning from the first nucleotide of the mRNA's 5′UTR. This program found hydrogen bonds between adenine (A) and uracil (U), guanine (G) and cytosine (C), G and U, and A and C. The distances between A and C were equal to those between G and C, A and U, and G and U. The numbers of hydrogen bonds in the G-C, A-U, G-U, and A-C interactions were found to be 3, 2, 1, and 1, respectively. The free binding energies of these nucleotide pairs were taken as the same values (i.e., 3, 2, 1, and 1, resp.) [12, 13].

## 3. Results and Discussion

### 3.1. Features of miR-619-5p, miR-5096, miR-5585-3p, and miR-5095

The binding powers between the 2,578 tested hsa-miRNAs and the mRNAs of 12,175 human genes were calculated. Some of these miRNAs have greater numbers of target genes than others. For example, miR-619-5p, miR-5095, miR-5096, and miR-5585-3p were found to be capable of binding more 600 genes each with value Δ*G*/Δ*G*_*m*_ ratios of 90% or more. These miRNAs were termed unique miRNAs (umiRNAs). Additionally, the binding sites for these unique miRNAs were unusually located in the mRNAs. miR-619-5p, miR-5095, miR-5096, and miR-5585-3p have different miRNA binding site origins, lengths, quantities, and miRNA binding site properties, among other features. Some characteristics of these unique miRNAs are outlined below.

With a length of 22 nucleotides (nt), miR-619-5*р* is coded in an intron of the slingshot protein phosphatase 1 host gene (*SSH1*), which is located on chromosome 12. We found that miR-619-5*р* has 1811 binding sites on 1215 target mRNAs. Of those, 1772 miR-619-5*р* binding sites are located in 3′UTRs, 26 sites are located in 5′UTRs, and 13 sites are located in CDSs. The mRNAs of 197 genes have completely complementary binding sites for miR-619-5*р*. The mRNAs of 27 genes have four binding sites. Seven genes have five binding sites, and the mRNAs of the* CATAD1, ICA1L, GK5, POLH, *and* PRR11* genes have six miR-619-5*р* binding sites. The mRNAs of the* OPA3 *and* CYP20A1 *genes have eight and ten binding sites, respectively. All of these sites are located in 3′UTRs.

With a length of 21 nt, miR-5096 is coded in an intron of the BMP2 inducible kinase host gene (*BMP2K*), which is located on chromosome 4. We found that miR-5096 has 997 binding sites on 832 target mRNAs. Of these, 984 miR-5096 binding sites are located in 3′UTRs, nine sites are located in 5′UTRs, and four sites are located in CDSs. The mRNAs of 42 genes have completely complementary binding sites for miR-5096. The mRNAs of the* IP09* gene have four binding sites. The* PRR11* gene has five binding sites. The mRNAs of the* OPA3 *and* CYP20A1 *genes have six and eleven miR-5096 binding sites, respectively. All of these sites are located in 3′UTRs.

With a length of 22 nt, miR-5585-3p is coded in an intron of the transmembrane protein 39b host gene (*TMEM39B*), which is located on chromosome 4. We found that 725 target gene mRNAs have 844 binding sites for miR-5585-3p. Nine of these binding sites are located in 5′UTRs, two sites are located in CDSs, and 833 sites are located in 3′UTRs. The mRNAs of the* CYP20A1 *and* GPR155* genes each have four binding sites.

With a length of 21 nt, miR-5095 is coded in an intron of the sterol carrier protein 2 host gene (*SCP2*), which is located on chromosome 1. We found that 655 target gene mRNAs have 734 binding sites. Fourteen of these binding sites are located in 5′UTRs, eight sites are located in CDSs, and 712 sites are located in 3′UTRs. The mRNAs of two genes have completely complementary binding sites for miR-5095. The mRNAs of the* OPA3 *and* SPN* genes each have four binding sites.

### 3.2. miRNA Binding Sites in 5′UTRs, CDSs, and 3′UTRs

**Figure 1:**
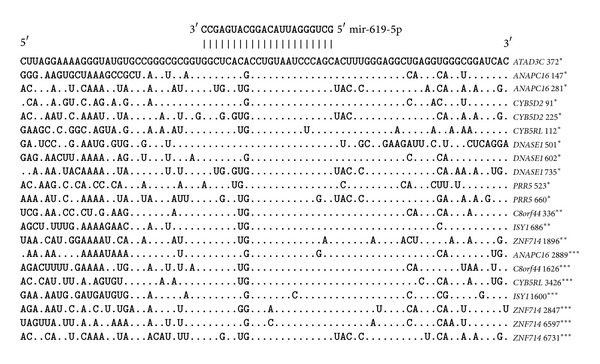
miR-619-5p binding sites in the 5′UTRs, CDSs, and 3′UTRs of human genes. Note: for Figures 1 and 2, the symbols ∗, ∗∗ and ∗∗∗ indicate the position of the origin of the miR-619-5p binding site from the first nucleotide of the 5′UTR in the 5′UTRs, CDSs, and 3′UTRs, respectively.

The miR-619-5p, miR-5095, miR-5096, and miR-5585-3p binding sites in the 5′UTRs, CDSs, and 3′UTRs of several genes were predicted using the MirTarget program. Multiple miRNA binding sites were revealed to be in the 5′UTRs of several genes. For example, miR-619-5*р* has two binding sites in each of the 5′UTRs of the* ANAPC16, CYB5D2*, and* PRR5* mRNAs and three binding sites in the* DNASE1* mRNA (Figure 1).

The mRNAs of some genes have binding sites for miR-619-5p, miR-5095, miR-5096, and miR-5585-3p within their 5′UTRs and 3′UTRs or CDSs and 3′UTRs. For example, the 5′UTRs and 3′UTRs of the* ATAD3C* and* CYB5RL *genes have miR-619-5p binding sites. The CDSs and 3′UTRs of the* C8orf44, ISY1*, and* ZNF714* genes have miR-619-5p binding sites.

**Figure 2:**
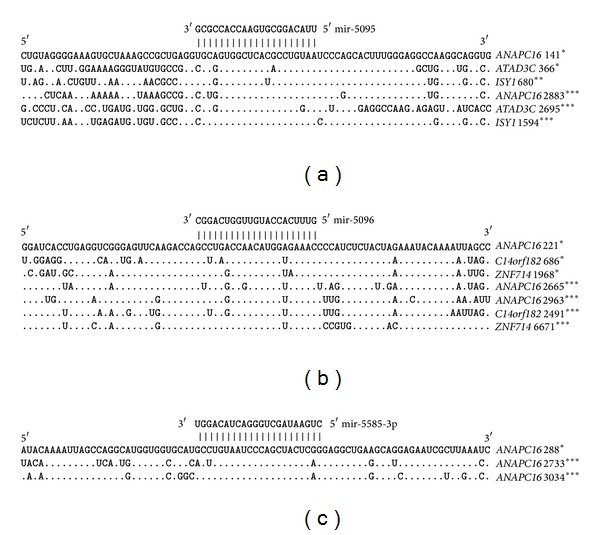
miR-5095, miR-5096, and miR-5585 binding sites in the 5′UTRs, CDSs, and 3′UTRs of human genes.

The 5′UTR and 3′UTR of the* ANAPC16* gene have miR-5095, miR-5096, and miR-5585-3p binding sites (Figure 2). The 5′UTR and 3′UTR of the* ATAD3C* gene have miR-5095 and miR-619-5p binding sites. The 5′UTRs and 3′UTRs of the* C14orf182* and* CYB5RL *genes have miR-5096 and miR-619-5p binding sites, respectively.

miR-5095 and miR-619-5p binding sites were found in the CDS and 3′UTR of the* ISY1* gene. The CDS and 3′UTR of the* ZNF714* gene have binding sites for miR-5096 and miR-619-5p, and the* C8orf44 *mRNA has only an miR-619-5p binding site.

The nucleotide sequences of the miR-619-5p binding sites mRNAs of the OPA3 and SPN genes each have four binding locate in the CDSs of the* C8orf44, ISY1*, and* ZNF714* genes'. The sites code for the following oligopeptides:

 
ENHWKGRA **RWLMPVIPALWEA** K **AG** R **S** *C8orf44,*
 
LFEKERQV **RWLMPVIPALWEA** E **AG** G **S** *ISY1,*
 
KHRKIQQGMV **AHACNPN** TLRGLGEQI *ZNF714.*

The first two oligopeptides are coded in one open reading frame (ORF), and the amino acids the miR-619-5p binding site codes for are highly conserved (highlighted in bold). The homologous oligonucleotide of the miR-619-5p binding site in* ZNF714* mRNA codes for an oligopeptide in other ORF. The presence of miR-619-5p binding sites in the CDSs of three genes with different functions and the evolutionary conservation of these sites testify to the importance of the role of miRNA in the regulation of the expression of these genes. The nucleotide sequences of the parts of the* C8orf44, ISY1*, and* ZNF714* mRNAs that contain the miR-619-5*р* binding sites in the CDSs are homologous among themselves and homologous with the binding sites located in the 5′UTRs and 3′UTRs.

In mRNA oligonucleotides, the miR-619-5*р*, miR-5095, miR-5096, and miR-5585-3p binding sites and the nucleotide sequences immediately downstream of the binding sites are highly homologous. It is possible that these downstream sequences are binding sites for other miRNAs. The sequences of the 5′UTRs, CDSs, and 3′UTRs upstream of the miR-5096 and miR-5585-3p binding sites are also homologous, likely for the same reason. The sequences of the mRNAs upstream of the miR-5095 binding sites are not homologous, and no other miRNA binding sites are identified in these sequences. The 10-nucleotide sequences upstream of the miR-619-5*р* binding sites contain homologous nucleotide sequences because they are the miR-5095 binding sites.

### 3.3. Multiple miRNA Binding Sites in the mRNAs of Target Genes

**Figure 3:**
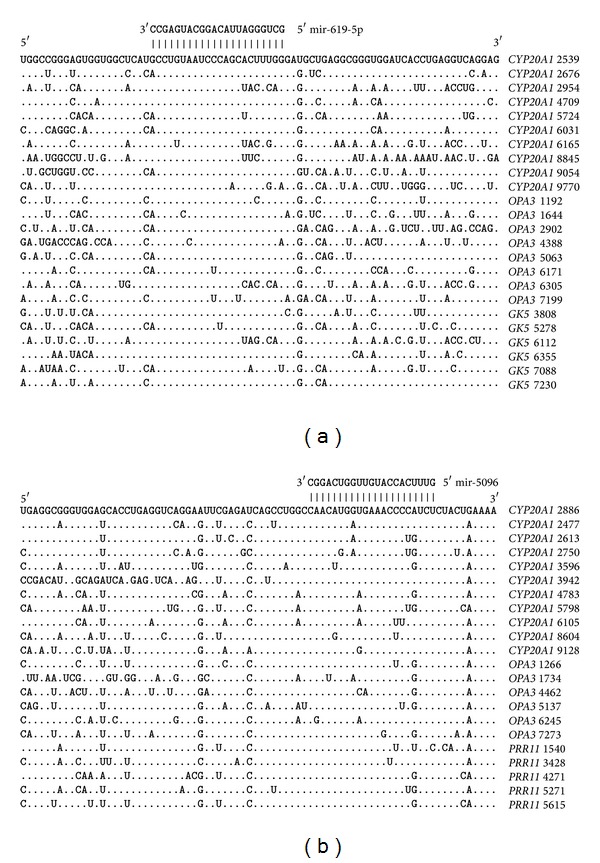
The nucleotide parts of 3′UTRs having multiple miR-5095 (a) and miR-5585-3p (b) binding sites. Note: ∗—one adenine was deleted before cytosine.

**Figure 4:**
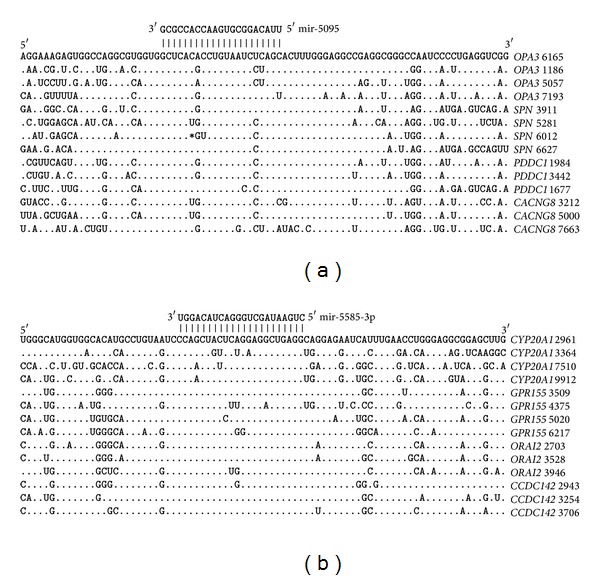
The nucleotide parts of 3′UTRs having multiple miR-619-5p (a) and miR-5096 (b) binding sites.

The mRNAs of some genes have multiple umiRNA binding sites. The nucleotide sequences with lengths of 95 nt that contain multiple miR-619-5p, miR-5096, miR-5095, and miR-5585-3p binding sites are given in Figures 3 and 4. These results testify to the high degree of homology between the umiRNA binding sites in the mRNAs of different genes. In addition to these binding sites, many other nucleotide sequences of the mRNAs are also homologous. It is possible that the nucleotide sequences adjacent to the binding sites are binding sites for other miRNAs.

### 3.4. The Arrangements of the Locations of umi-RNA Binding Sites

**Figure 5:**
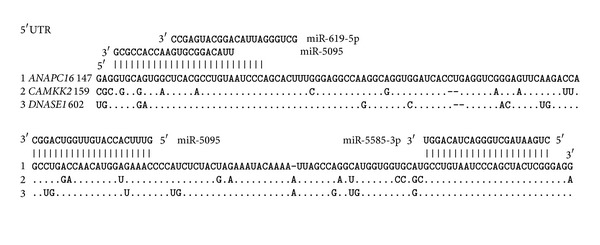
miR-619-5p, miR-5095, miR-5096, and miR-5585-3p binding sites located in 5′UTRs.

The mRNAs that were targeted by miR-619-5p, miR-5096, miR-5095, and miR-5585-3p were established. The 5′UTRs of three target genes contain these miRNA-binding sites (Figure 5). The degree of homology of the nucleotide sequences in these genes is high not only in the binding sites of the studied miRNAs but also across all mRNA 150 nt sequences. The distance between the miR-5095 and miR-5096 binding sites is 57–59 nt and that between the miR-5096 and miR-5585-3p binding sites is 46-47 nt. The miR-5095 and miR-619-5p binding sites partially overlap.

**Figure 6:**
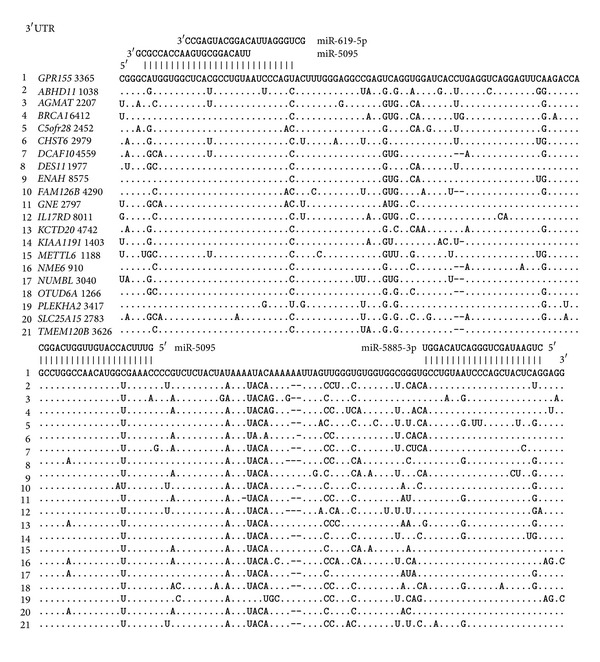
miR-619-5p, miR-5095, miR-5096, and miR-5585-3p binding sites located in the 3′UTRs.

The greatest numbers of miR-619-5p, miR-5096, miR-5095, and miR-5585-3p binding sites are located in the 3′UTRs, and it is, therefore, possible that many target genes have umiRNAs binding sites. The data about the locations of the miR-619-5p, miR-5096, miR-5095, and miR-5585-3p binding sites and the degrees of homology of the corresponding nucleotide sequences in the mRNAs of 21 genes are presented in Figure 6. The distances between the miR-5095 and miR-5096 binding sites are all 57–60 nt. The distances between the miR-5095 and miR-5096 binding sites in the mRNAs of 78 genes averaged 58.6 ± 0.9 nt. Thus, the distances between miR-5095 and miR-5096 binding sites are highly conserve. The distances between the miR-5096 and miR-5585-3p binding sites are all 46–49 nt. The distances between the miR-5096 and miR-5585-3p binding sites in the mRNAs of 325 genes average 47.3 ± 1.1.

The degree of homology of the nucleotide sequences containing the miR-619-5p, miR-5096, miR-5095, and miR-5585-3p binding sites is high. These areas contain binding sites for miRNAs other than the studied umiRNAs. Other miRNA binding sites are not present in all genes, and these binding sites have lower affinities (data not shown). It is possible that there are conserved domains in the nucleotide sequences of mRNAs.

### 3.5. Variability in the Arrangement of umiRNA Binding Site Locations

**Figure 7:**
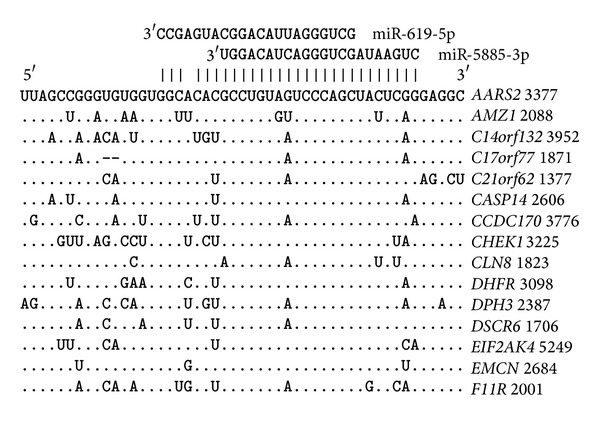
3′UTRs with miR-619-5p binding sites upstream of the miR-5585-3p binding sites. Note: Symbol “≈” indicates nucleotide sequence equaled to 84 nt, which is not shown here.

The miR-619-5p binding site is located at a distance of six nucleotides downstream of the beginning of the miR-5095 site in the majority of genes containing arranged umiRNA sites. However, in another group of mRNAs, the beginnings of the miR-619-5p binding sites are located at distances of seven nucleotides upstream of the miR-5585-3p binding sites (Figure 7).

**Figure 8:**
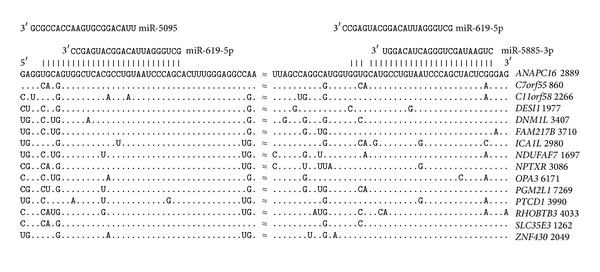
3′UTRs in which the miR-619-5p binding site was downstream of the miR-5095 binding site and upstream of the miR-5585-3p binding site.

There is another group of genes in which the miR-619-5p binding sites are downstream of the miR-5095 sites and upstream of the miR-5585-3p sites (Figure 8). The distances between the positions of the two miR-619-5p binding sites in the mRNAs of these genes are constant at 112 nt. The nucleotide sequences of the mRNAs with miR-619-5p, miR-5095, and miR-5585-3p binding sites are highly homologous, which testifies to the strength of the selection pressure on these nucleotide sequences.

## 4. Conclusion

The detection of a large number of binding sites of miR-619-5p, miR-5095, miR-5096, and miR-5585 in the mRNAs of the genes studied here presumably indicates new functional opportunities. It is possible that these umiRNAs are coordinators of gene expression that participate in many major biological processes. The influences of miRNAs on the expression of genes that code for transcription factors [14, 15] and proteins that participate in the cellular cycle [3], apoptosis [4], stress responses [16], and so forth, have previously been shown.

This study established that the binding sites of umiRNAs and miRNAs are in 3′UTRs, CDSs, and 5′UTRs. Highly conserved miR-619-5p, miR-5095, miR-5096, and miR-5585 binding sites in a large number of genes indicate the emergence of these sites in the early stages of human evolution. We have shown previously that miRNA binding sites located in CDS are conserved in target orthologous genes of organisms that diverged some hundred million years ago [17, 18].* SSH1, BMP2K, TMEM39B*, and* SCP2* host genes and target genes of miR-619-5p, miR-5095, miR-5096, and miR-5585 have independent evolution origin. Revealed miRNA binding sites have arranged localization in mRNA of multiple target genes participating in different metabolic processes. Host genes often coexpress with their intragenic miRNAs; therefore, an expression of* SSH1, BMP2K, TMEM39B*, and* SCP2* genes can be connected with target genes via their miRNAs. Thus, it predicted that host genes and target genes are interconnected between themselves. Arranged localization of binding sites suggests an interconnected evolution of miRNAs and their target genes. Conservatism of the arranged localization of miRNA binding sites in 3′UTRs, 5′UTRs, and CDSs also demonstrates the common origin of the binding sites and the evolution of relationship of miRNAs with their target genes.
